# A single-film fiber optical sensor for simultaneous measurement of carbon dioxide and relative humidity

**DOI:** 10.1016/j.optlastec.2021.107696

**Published:** 2022-03

**Authors:** LiangLiang Liu, Stephen P. Morgan, Ricardo Correia, Serhiy Korposh

**Affiliations:** Optics and Photonics Group, Faculty of Engineering, University of Nottingham, University Park, Nottingham NG7 2RD, UK

**Keywords:** Optical fiber sensor, Fabry–Pérot interferometer, Colorimetric change, Relative humidity, Respiration, Multiparameter sensing

## Abstract

Colorimetric measurement is a versatile, low-cost method for bio-/chemical sensing and that has importance in biomedical applications. General carbon dioxide (CO_2_) sensors based on colorimetric change of a pH indicator report only one parameter at a time and are cross-sensitive to relative humidity (RH). This work describes a novel optical fiber sensor with a thin film on the distal end of the fiber, combining colorimetric measurement and a white light Fabry–Pérot interferometer (FPI) for the simultaneous measurement of CO_2_ and RH. The CO_2_ sensitive dye ion-pair: thymol blue and tetramethylammonium hydroxide are encapsulated inside organically modified silica forming an extrinsic FPI cavity (refractive index of 1.501 ± 0.02 and thickness of 5.83 ± 0.09 μm). The sensor reversibly responds to 0–6% CO_2_ and 0–90% RH with negligible cross-sensitivity and allows measurement of both parameters simultaneously. A sensitivity of ∼0.19 nm/%RH is obtained for RH measurement based on the wavelength shift of the FPI and there is a polynomial correlation between the average intensity of selected wavelengths and the concentration of CO_2_. The applicability of the sensor is demonstrated by measuring the CO_2_ and RH exhaled from human breath with a percent error of 3.1% and 2.2% respectively compared to a commercial datalogger. A simulation model is provided for the dye-encapsulated FPI sensor allowing simulation of spectra of sensors with different film thicknesses.

## Highlights

• Simultaneous measuring CO_2_ and RH with a single-film functionalised fibre optic probe.
• A combination of colourimetric measurement and the Fabry–Pérot interferometry.
• Measuring RH in the range of 0–90% and CO_2_ concentration of 0–6%.

## 1 Introduction

Carbon dioxide (CO_2_) in the human body is produced by cellular metabolism, and is transported in a dissolved format (H_2_CO_3_) via blood flow and diffuses into alveoli at the lungs where it is subsequently expelled from the body with each breath [1]. In some pathological conditions such as asthma and chronic obstructive pulmonary disease, the functionality of the lungs is affected and, as a result, CO_2_ cannot be fully released through breathing. The retention of CO_2_ in the blood can cause respiratory acidosis which can be life-threatening [2]. End-tidal CO_2_ (ETCO_2_) is the measurement of CO_2_ at the end of each breath and used to evaluate those pulmonary diseases. It is also used to verify the correct placement of an endotracheal tube during the procedure of assisted ventilation [3]. Humidity is important in the healthcare environment as not only does it affect the operation of expensive electrical equipment due to the static spark, but more importantly, affects the comfort and health of the patient [4], [5], [6]. Exhaled humidity from the breath is also an important parameter that needs to be measured in spirometry for diagnosis of pulmonary deficiencies [7], [8], especially in the determination of pulmonary oxygen uptake during anaesthesia [9].

A low-cost sensor that is compatible with the healthcare environment will make a contribution to improve health. Fiber optical sensors (FOS), due to the numerous advantages of light over electronic systems, have drawn a lot of research interest in a range of applications including healthcare [10]. An interesting application is monitoring relevant parameters during MRI scanning where it is challenging to utilise conventional electrical sensors due to strong electromagnetic interference.

General approaches of optical fiber sensing of CO_2_ use pH dyes such as thymol blue, phenol red and α-naphtholphthalein [11], [12], [13], [14], [15] or fluorescent compounds such as 1-hydroxy-3,6,8-pyrenetrisulfonic acid trisodium salt (HPTS) [16], [17], [18] with quaternary ammonium hydroxide which convert gaseous CO_2_ into a dissolved format leading to a change of pH. Such pH dyes or fluorescent compounds change their spectral properties according to the degree of the pH change induced by CO_2_. The dyes or fluorescent compounds are usually doped into a matrix film such as a porous silica [17] or a polymer film [14] along with the quaternary ammonium hydroxide on the optical fiber in a region where light interacts within the film such as the tip or cladding removed region. One common problem reported is cross-sensitivity to humidity [11], [12], [15], and the sensors are recommended to be applied in an environment with controlled humidity level, which is a major practical issue for the implementation of such sensors. The reported sensors can only measure a single parameter which is CO_2_ and does not allow the specific measurement of RH. The capability of measuring more than one parameter such as humidity simultaneously by using a single sensor will provide a great advantage for humidity compensation, miniaturisation and versatility. An interesting optical fiber multi-sensing CO_2_ sensor is reported by Wu et al. in which the dye-free polymers are in situ optically printed on the end surface of a multicore optical fiber, different polymers on the top of each core constitute an individual sensor for simultaneously sensing of CO_2_ and temperature [19]. Other interesting approaches of optical fiber multi-sensing include encapsulation of multiple fluorescent dyes on the fiber tip [20], the two-film structure on the fiber tip [21].

These approaches are relatively complex and at present are not compatible with simple, low cost manufacture and therefore widespread implementation. A potential candidate for optical fiber sensing of CO_2_ is to use pH indicators with the organically modified silica (Ormosil) as the matrix film [11], [15]. Properties of the matrix film such as polarity and porosity vary based on the molar ratio of the mixtures, reaction temperature as well as drying condition and these can affect the sensing performance through the permeability of the CO_2_ as well as the cross-sensitivity to humidity. This makes relating the CO_2_ response difficult to interpret when humidity changes. A method of sensing CO_2_ with low RH cross talk is therefore highly desirable.

In this work, a reflection mode fiber optic CO_2_ sensor is introduced that combines the colorimetric method and Fabry–Pérot interferometer (FPI) technique to measure CO_2_ and RH simultaneously. In this case Ormosil not only functions as the matrix film for dye encapsulation but is also used for sensing of RH that renders the multi-sensing capability in a single film. The sensor is fabricated by dip coating a CO_2_ sensitive film which contains thymol blue and phase transfer additive doped inside of the porous Ormosil film on the tip of an optical fiber. The coated film acts as an extrinsic FP cavity for the FPI with the thickness controlled by the dip coating process. The tip-based fiber sensor probe provides a smaller dimension (125 μm diameter, ∼6 μm thickness) in the sensor element region and flexibility in application. Moreover, the proposed hybrid tip sensor with a single film allows monitoring two parameters (CO_2_ and RH) simultaneously which to the best of our knowledge is the first time this has been reported. The developed FOS exhibits a wide detection range of 0–6% CO_2_ and 0–90% RH and demonstrates a comparable performance in measuring the two parameters from human breath to a commercial datalogger. Such a tip-based extrinsic FPI with dye encapsulation can be applied in theory to any colorimetric sensing technique for either sensing of dual parameters or humidity compensation. Theoretical equations are derived for the dye interferometer allowing the simulation of spectra of different thicknesses.

## 2 Method

### 2.1 Sensing mechanism for CO_2_ and RH

**Fig. 1:**
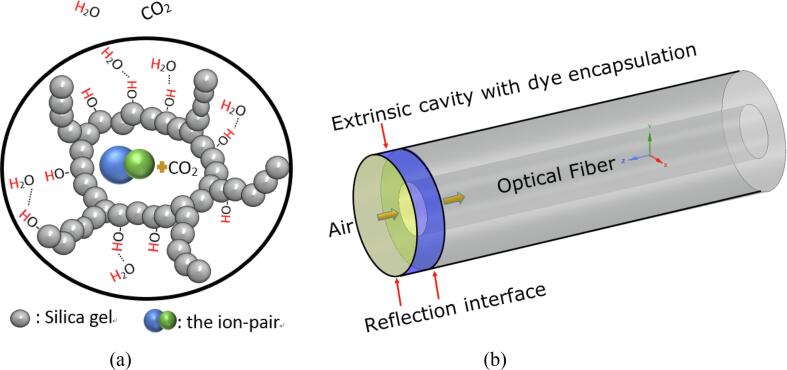
Schematic illustration of: (a) the mesporous film with encapsulation of sensing element. (b) the FOS sensor with an external film coated on the tip forming a Fabry–Pérot cavity.

The TEOS and MTEOS (listed in Materials of Supplementary information) undergo hydrolysis and condensation during the Sol-gel reaction resulting in a cross-linked silica network with the formation of siloxane bonds (≡Si—O—Si≡) between silanol groups (—Si—OH). Thymol blue (HT) and TMAH (QOH) are the dye and phase transfer for sensing CO_2_ respectively, are physically trapped inside the network in format of ion-pairs ($Q^{+}T^{-}\cdot xH_{2}o$) (illustrated in Fig. 1a). CO_2_ diffuses into the film through the mesoporous structure of the film and reacts with the dye ion-pair producing a colour change. The colour transmission of the above process can be explained by:

(1) $$\mathrm{QOH}+HT\left(red\right)⇌{\{Q}^{+}T^{-}\cdot xH_{2}o\}\left(blue\right)$$

(2) $${\{Q}^{+}T^{-}\cdot xH_{2}o\}\left(blue\right)+CO_{2}⇌Q^{+}HCO_{3}^{-}\cdot \left(x-1\right)H_{2}o+HT\left(yellow\right)$$

The colour transits from red to blue as the $OH^{-}$ ion of phase transfer (TMAH) deprotonates the thymol blue forming ion pairs (Eq. (1)). While CO_2_ reacts with the hydrated water molecule of ion pairs, it produces protons that protonate the thymol blue ($T^{-}$) leading to a colour change towards yellow (Eq. (2)).

The dye encapsulated sol–gel film coated on the tip of the optical fiber forms an extrinsic Fabry–Pérot cavity with a length of few micrometres (～6 μm measured from Fig. 4b) as illustrated in Fig. 1b. The light transmits from the fiber into the film and from the film into air; reflectance occurs at each interface (differential refractive index medium) on its pathway.

Due to the differential phase of reflected light between the interface of the optical fiber with the coated film and the interface of the coated film with air, FPI fringes can be observed when the thickness of the coated film is less than the coherence length of the light source. The normalised intensity returning from the dye encapusulated film coated tip ($I_{\lambda })$ can be simulated by adapting modified FPI equations from [22] with the Beer-Lambert law [23] as expressed below (the detailed equation derivation is attached in Supplementary information):

(3) $$I_{\lambda }=R_{1}+K^{2}{\left(1-R_{1}\right)}^{2}R_{2}+2\sqrt{R_{1}R_{2}}K\left(1-R_{1}\right)cos\left(\frac{4\pi }{\lambda }nL-arctan\left(\frac{2\lambda L}{\pi \omega_{0}^{2}}\right)\right)$$

(4) $$K={\left[1+\frac{4L^{2}\lambda^{2}}{\pi \omega_{0}^{4}}\right]}^{-1/4}\left(1-A_{1}\right)e^{-aL}$$

(5) $$a=2.303c\varepsilon_{\lambda }=2.303A/L$$

where $R_{1}$ and $R_{2}$ are the reflections from the two interfaces in Fig. 1, the content of cosine function is the phase difference, λ is the wavelength of incident light, *L* is the cavity length and *n* is the refractive index of the cavity material, K is the total loss factor of the sensor head, $\omega_{0}$ is beam waist diameter, $A_{1}$ is the transmission loss factor of cavity, a is the absorption coefficiency of the dye (thymol blue) and is associated with the extinction coefficient ($\varepsilon_{\lambda }$) and the dye concentration (c) within the cavity (Beer-Lambert law). The above equations allow us to simulate the reflection spectrum which can be converted into absorption spectrum by applying the logarithmic function ($A_{\lambda }=\log_{10}\left(1/I_{\lambda }\right)$). When a perturbation is introduced to the FPI such as a change of refractive index, the phase difference changes in proportion to the optical path length of the interferometer. The change of phase difference is observed as a shift of the wavelength of the interference fringes in the reflection spectrum, and the degree of the perturbation can be quantitatively related to the shift in wavelength. When increasing the atmospheric RH level, the water molecules are adsorbed by the unreacted hydrophilic silanol group (—Si—OH) of the Ormosil matrix film via hydrogen bonds (illustrated in Fig. 1a), leading to a change of refractive index. As a result, the phase difference of the two interference beams of the FPI is changed and the interference fringes undergo a shift.

The thickness of sensing film can be calculated:

(6) $$L=\frac{\lambda_{1}\lambda_{2}}{2n\left(\lambda_{2}-\lambda_{1}\right)}$$

where *L* is the thickness of the film (cavity length), *n* is the refractive index of the film, $\lambda_{1}$ and $\lambda_{2}$ are the central wavelength of two adjacent inteference peaks.

### 2.2 Method

#### 2.2.1 Sensor preparation

The sensor fabrication process is provided in the Supplementary information.

#### 2.2.2 Experimental set-up

**Fig. 2:**
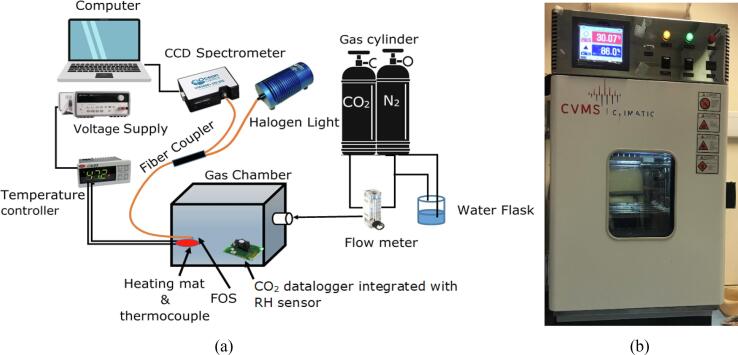
(a) Schematic illustration of the experimental setup for the calibration of FOS. (b) The environmental chamber for measuring RH up to 90%.

The FOS is calibrated in a gas chamber (15 cm (L) × 13 cm (W) × 8 cm (H)) by using the set-up in Fig. 2a in which a commercial CO_2_ datalogger integrated with a humidity and temperature sensor (K33, CO_2_ Meter) is placed inside the chamber as a reference sensor. A temperature controlled heating mat constructed with a 50 mm heating mat, a J type thermocouple and a controller (IR33, Carel) is used to modulate the temperature of the FOS which sits on the top of the mat and placed inside of the chamber. The concentration of CO_2_ inside the chamber is regulated by mixing of N_2_ and CO_2_ gas through a flow meter. Humidity is generated through humidifying the N_2_ gas flowing into the chamber (this method only provides RH up to 80%). A halogen light (HL-2000, Ocean Optics) and a CCD spectrometer (USB 2000, Ocean Optics) are used to obtain the spectral information of the FOS.

The FOS is also tested in an environmental chamber (Bench Top, C-TH40-20/1, Climatic Ltd) for the RH calibration at a high level (up to 90%) (illustrated in Fig. 2b). The environmental chamber was programmed to continuously increase the RH from 50% to 100% with temperature stabilised at 25 °C.

**Fig. 3:**
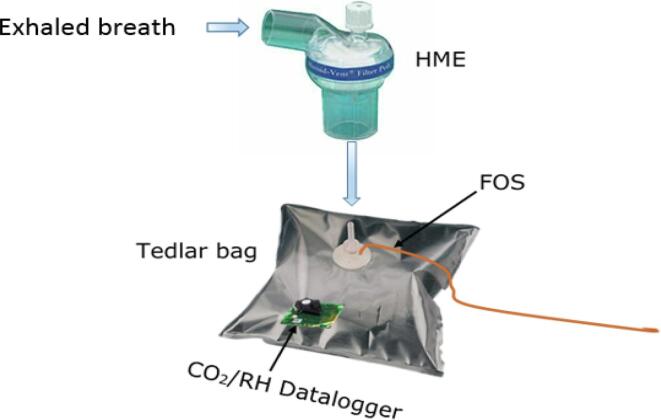
Schematic experimental set-up for the breath gas measurement.

Breath sample measurement is conducted by breathing into a Tedlar bag (2 L, Sigma Aldrich) which has the FOS and commercial datalogger placed inside to record the CO_2_ and RH change before and after breathing (Fig. 3). A heat moisture exchanger (HME) (Clear-Therm, Intersurgical Ltd), typically used in mechanical ventilators [24], was applied behind the mouthpiece to prevent the condensation of humidity after the exhaled gases leave the human body (37 °C) to ambient temperature (22 °C).

**Fig. 4:**
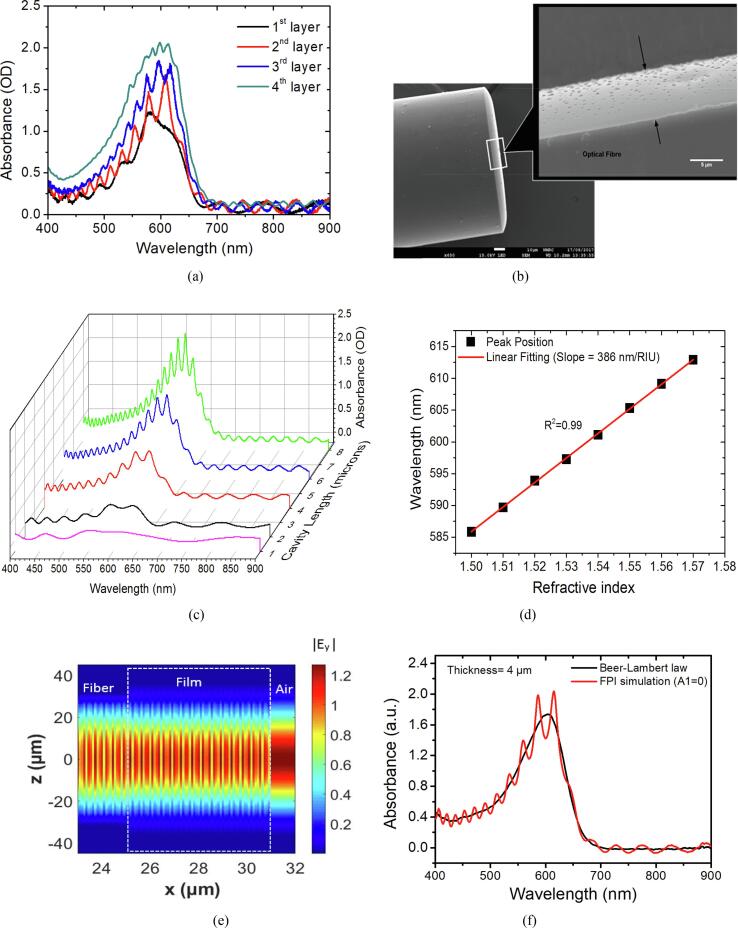
(a) Absorption spectrum of FOS after the n^th^ layer coating (n = 1, 2, 3, 4); each spectrum is taken after 2 mins of each coating. (b) SEM image of three layers sensing film after drying in N_2_ for 1 day on the tip of an optical fiber; the coated film is shown between two black arrows, and the thickness is measured about 5.95 μm (scale bar = 5 μm). (c) Simulated absorption spectrum of different cavity lengths. (d) The simulated RI response of a FPI sensor with cavity length, L = 6 µm. (e) The calculated electric field profile within the coated fibre. The mode is fundamental mode with simulated wavelength of λ = 700 nm and cavity length, L = 6 µm. (f) The comparison of the simulated reflection spectrum with spectrum calculated with the Beer-Lambert law.

#### 2.2.3 Sensor characterisation

The sol–gel film encapsulated with thymol blue and TMAH is deposited on a silicon wafer via dip coating and measured under a Spectroscopic Ellipsometer (alpha-SE, J.A.Woollam) after drying in N_2_ to obtain the refractive index value. The coating film on the fiber tip and its thickness are imaged and measured by using SEM (JEOL 7100F). The thickness of the film is also calculated from Eq.(6) based on the measured refractive index.

For calibration of CO_2_, the measurement chamber shown in Fig. 2a is initially filled up with 100 % N_2_ and then the concentration is built up step-by-step by regulating the CO_2_ flow and allowing each step to stabilise for at least 5 min. The reflection spectra of the FOS and readings from the commericial CO_2_ datalogger are recorded simultaneously. The average normalised intensity value of the wavelengths between 596 nm and 617 nm (peak-to-peak), corresponding to the absorption band of the thymol blue, is utilised as the optical signal for calibration with the commercial CO_2_ datalogger. The CO_2_ calibration curve is obtained by averaging the optical signal during the stabilised period (the last 5 mins of each step) against the average concentration of CO_2_ achieved from the datalogger in the same period. The reported percent error represents the difference between the measurement value of a measurand by using the FOS and the reference sensor, divided by the reference value reported from the reference sensor.

For humidity calibration, the CO_2_ and RH level is initially brought close to zero by filling the measurement chamber with dry N_2_, and then the humidified N_2_ flows into the chamber to gradually increase the RH level step-by-step (up to the ∼ 80% limit of the experimental set-up, Fig. 2a) while maintaining CO_2_ at a low (<20 ppm) level. The RH calibration above 80% was conducted in the environmental chamber (Fig. 2b) by programming a RH change from 50% to 100% and then from 100% to 50% for 4 repeating cycles with the temperature maintained at 25 °C. The wavelengths of the interference peaks were used to calibrate the RH level by comparing the central wavelength with the RH reading from the commercial humidity datalogger.

The effects of temperature are invesitgated by gradually increasing the temperature of the heating mat from 20 °C to 40 °C with an interval of 5 °C at different concentrations of CO_2_. The temperature fluctuation is < 1 °C for each setting.

To produce a rapid change in CO_2_ in order to measure the sensor response time, the fiber is inserted into the measurement chamber filled with ∼6% of CO_2_, then there is a pause until the signal is stable and then the cap is opened to release the CO_2_ back to ambient conditions. For creating a high level of humidified environment, 50 ml of distilled water was poured into a petri dish and placed inside of the chamber until the RH level inside of the chamber is stabilised (after approximately 1 h, the RH value was ∼ 76%). The same insertion and open cap process used for CO_2_ is then repeated to obtain the RH response time. The response time is defined as the time between 10% and 90% of the signal change when the signal changes from one stable value to another in response to the presence of the analyte.

## 3 Results

### 3.1 Characteristic of sensor

Fig. 4a shows the absorption spectrum after each coating cycle on the tip of the optical fiber during the coating process. The absorption window, corresponding to the absorption band of thymol blue, appears between 500 and 700 nm and the FPI fringes appear in each spectrum. The absorption value increases after each coating cycle due to the increased optical pathlength according to the Beer-Lambert law. The number of fringes increases after each layer coating as a result of the increase of cavity length (thickness of the film). The visibility of fringes decreases at the fourth layer, and the fringes disappear after further increase of the number of coating cycles.

The absorption value between 500 and 700 nm decreases after drying for 24 h due to decomposition of base catalysts during the evolution of the sol–gel film; One coating cycle on the tip of the fiber is observed to have a very low absorbance after drying (shown in Fig. S.2) and is thus not suitable for CO_2_ measurement due to insufficient colour change. Multiple coating cycles provide thicker films with more dye encapsulated resulting in a higher absorbance value and measurable colour change after exposure to CO_2_. Three layers are used in this work as the film has sufficient absorption as well as high visibility of fringes. The refractive index of the sensing film after drying is measured as 1.501 ± 0.02 via ellipsometry. The thickness is calculated as 5.83 ± 0.09 μm from Eq. (6) and agrees well with the 5.95 μm measured with SEM in Fig. 4b.

A FPI sensor simulation model is built with the measured parameters (i.e. film RI and thickness) by using equations described in Section 2.1. The extinction coefficient of the dye at deprotonated status are obtained with UV–Visable absorption spectroscopy. Fig. 4c shows the absorption spectrum of the optical fiber hybrid FPI sensor with different cavity lengths ranging from 1 to 8 μm. The number of fringes increases with increase of cavity length and absorbance value at dye absorption region increases correspondingly. These spectral changes with increase of cavity length agree with experimental results in Fig. 4a. As increase of the RI of the film, the interference wavelength of the simulated spectrum shifts to a higher wavelength corresponding to a sensitivity of 386 nm/RIU at a initial wavelength around 585 nm. Fig. 4e illustrates electric field distribution (E_y_) of the fundamental mode along the coated fiber indicating FP interference occurs between the forward and backward (reflected) propagation. It is obtained by using *Lumerical MODE* (version: 7.15.2305), software that calculates light propagation with bidirectional eigenmode expansion (EME). To validate the above derived FPI equation for a tip-based optical fibre sensor. Its simulated absorption spectrum is plotted against the spectrum obtained by using Beer-Lambert law (Eq. (5)) in which the optical pathlength L is multiplied by two due to reflection mode. The result in Fig. 4f demonstrates that the absorption spectrum obtained via the proposed FPI dye sensor equation matches to its obtained via Beer-Lambert law equation but with the interference fringes added on it proofing the derived equation is valid for the dye based interferometer.

### 3.2 CO_2_ measurement using FOS

**Fig. 5:**
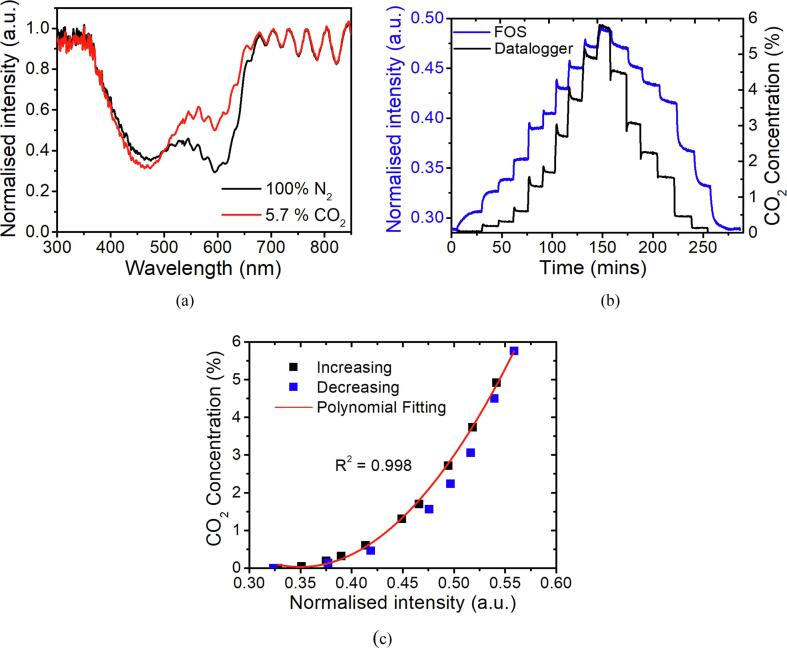
(a) Reflection spectrum of FOS in 100 % N_2_ and 5.7% CO_2_. (b) The signal response of FOS in comparison to the CO_2_ reading from a commercial datalogger against time. The intensity value is taken as the average intensity value between wavelengths of 596–617 nm. (c) Polynomial fitting of the data; each point is achieved by taking the average value of each stabilized step in (b), and error bar is smaller than the marker size;

Fig. 5a illustrates the reflection spectrum of the FOS in 100 % N_2_ and 5.7% CO_2_. Within the absorption window (λ = 350–670 nm), the normalised intensity value increases around 600 nm and decreases at 450 nm after exposure to CO_2_ as a result of protonation of the dye in Eq. (2). In this case, the colour of the film turns from blue (deprotonated status) towards yellow (protonated status). The position of the interference fringes remains unchanged during the test indicating no cross-talk between the fringe position and the concentration of CO_2_. This also suggests that there is no detectable refractive index change after the interaction of CO_2_. The average intensity value of the selected wavelengths (596–617 nm, wavelengths are between two adjacent interference peaks around the absorption peak) is extracted to compare with the CO_2_ reading from the commercial datalogger as shown in Fig. 5b. This is taken to avoid the intensity change of a single wavelength induced by the shift of fringes in the RH measurement and the average intensity is considered relatively immune to the shift of the wavelength. The normalised intensity value responds in proportion to the change of CO_2_ concentration following a polynomial relationship and demonstrating the responsitivity and reversibility of the FOS (Fig. 5c). Hysteresis is observed for the reverse trace which is likely caused by the incomplete release of CO_2_ during the recording time as it is a slow process. The response time and recovery time is calculated as 98 s and 418 s, respectively (shown in Fig. S.3). The sensor exhibits a longer recovery time than the commercial datalogger due to the low equilibrate time of the chemical reaction. The FOS was inserted into the CO_2_ chamber, and it was immediately exposed to CO_2_ atmosphere so that the calculated response time is considered as the sensor response time. The sensor recovery time was achieved by opening the chamber cap and allowing the CO_2_ to diffuse into the air and be diluted and the recovery time of FOS is considered to include the CO_2_ diffusion time. The RH level during the CO_2_ test was relatively stable at 19 ± 1.9%.

### 3.3 RH measurement by using FOS

**Fig. 6:**
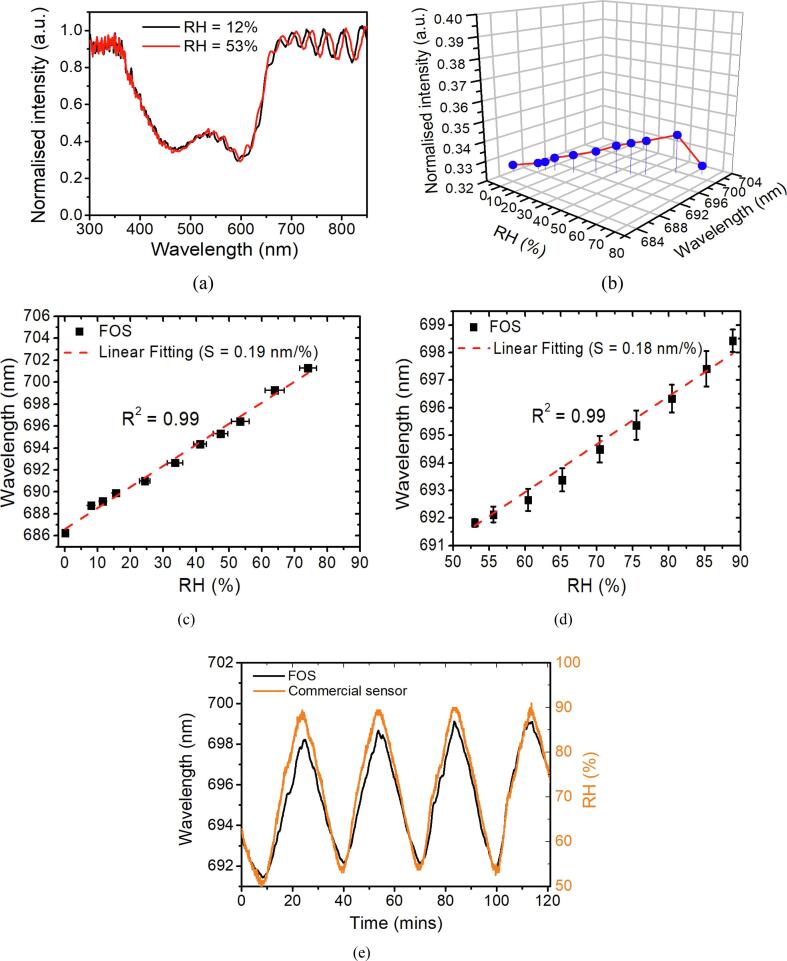
(a) Reflection spectrum of FOS at different RH levels. (b) The average intensity value and a peak position under different RH levels. (c) Linear fitting of the wavelength of FOS to the RH range from 0 up to 80%. (d) Linear fitting of wavelength against the RH for the range from 50% to 90% (e) Repeatability of RH measurement range from 50% to 90%.

The concentration of CO_2_ is under the limit of detection of the commercial datalogger (<20 ppm) during the RH test, thus the test environment can be treated as CO_2_ free. The interference fringes undergo a redshift after exposure to increasing levels of RH as illustrated in the reflection spectrum in Fig. 6a. This is due to increased film refractive index as demonstrated in Fig. 4d. The intensity value (described in Section 3.2) remains relatively stable as the RH level increases from 0% to ∼80% whereas a selected peak wavelength (around 686 nm) exhibits a gradual increase (illustrated in Fig. 5b). The intensity slightly increases as the concentration increases from 0% to 65%, this is due to the protonation of water molecules to alkalic thymol blue. The overall increase of the intensity caused by the 65% of RH is incorrectly equal to the CO_2_ concentration of 232 ppm according to the CO_2_ response in Fig. 5c. The small drop of the intensity when RH is above 70% is caused by the decrease of reflectivity of light due to adsorption of water molecules on the film-air interface. Thus, this relatively stable intensity value ensures the CO_2_ measurement has a negligible cross sensitivity to RH. Each peak of interference fringes shows the same trend of linear correlation to the RH level range from 0% up to ∼80% and among them the peak around 686 nm exhibits the highest sensitivity of 0.19 nm/1% RH (Fig. S.4). Therefore, the wavelength with initial position of 686 nm is tracked to indicate the RH change in comparison to the reading from the reference commercial datalogger. The peak wavelength as a function of RH is shown in Fig. 6c and d which are obtained from the set-ups in Fig. 2a and b to cover the whole range of RH. Although the RH setting in the environmental chamber was set up to 100%, the true RH seems saturated at around 90% only. The wavelength shift of the FOS shows good agreement with the RH change inside the environmental chamber. Fig. 6e shows the repeatability test of FOS when varying RH from 50% up to 90% and the wavelength of the FOS exhibits excellent reversibility and repeatbility to RH. The response and reverse time of the sensor for the RH from ambient level (∼33%) to 76% are calculated as 32 s and 56 s, respectively (Fig. S.5). The faster response time of RH than CO_2_ attributes to the faster diffusion speed of water molecule in the silica film due to its smaller molecular weight according to Knudsen diffusion [25].

### 3.4 Temperature effect on the FOS

**Fig. 7:**
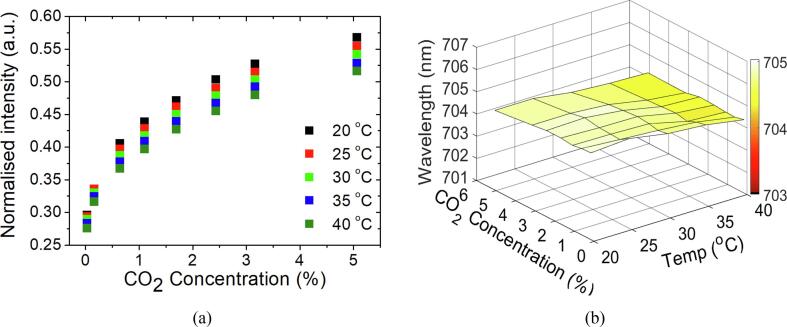
(a) The intensity of selected wavelength response to CO_2_ concentrations at different temperature. (b) The wavelength of the interference fringe at different concentration of CO_2_ and temperature.

It is also important to achieve low cross-sensitivity to temperature. Fig. 7a shows the correlation of intensity to different levels of CO_2_ at different temperatures from 20 °C to 40 °C. It shows that the sensor has a lower sensitivity at higher temperature and this phenomenon is more pronounced at higher concentrations (>0.5%). The intensity decreases approximately 6% when temperature increases from 20 °C to 40 °C at 0.03% of CO_2_ whereas it drops about 9% at 5% of CO_2_. Within 5 °C of temperature change, the percent error for measuring 5% CO_2_ is < 14%. The wavelength of the FPI fringes, on the other hand, does not change as the CO_2_ concentration increases as shown in Fig. 7b (the biggest change is ∼ 0.3 nm). It has a wavelength shift of < 0.6 nm as the temperature increases from 20 °C to 40 °C, which is about 3.3% less than the corresponding RH change. This change is probably caused by the change of RH inside of the chamber.

### 3.5 The use of FOS for measurement of CO_2_ and RH from human breath

**Fig. 8:**
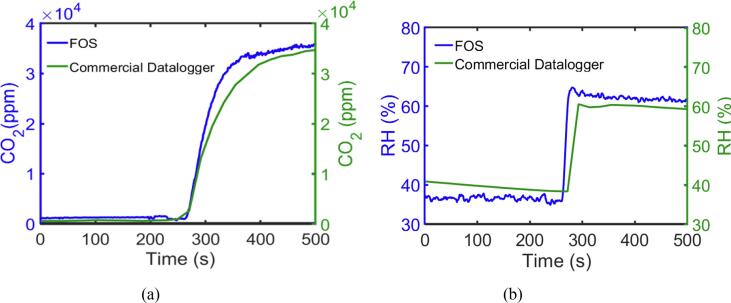
(a) The response of FOS to the CO_2_ from human breath; (b) The response of FOS to the RH from human breath.

Fig. 8 illustrates the simultaneous measurement of CO_2_ (Fig. 8a) and RH (Fig. 8b) from human breath by using the developed FOS compared to the commercial datalogger. The datalogger shows that the CO_2_ concentration inside the Tedlar bag increases from ambient level (ca. 0.08%) to 3.5% after breath and that the RH level increases from about 40% to 60%. The concentration for both parameters is less than the normal breath level (5% CO2, 90% RH), this is most likely caused by the dilution of breathing gas into the Tedlar bag as it was sealed with approximately 100 to 400 ml of air for supporting the commercial sensor and FOS. The measurement of the two parameters from the FOS has good agreement with the datalogger with a percent error of 3.1% for CO_2_ and 2.2% for RH. The signal of the rising part for both commercial datalogger and FOS includes the breathing process and the gas diffusion time.

## 4 Discussion

As the interference of an FPI sensor is dependent on the cavity length (Eq.(3), (4)), the film thickness of the proposed extrinsic FPI sensor needs to be precisely controlled in order to obtain the FPI. As white light interferometry is extremely sensitive to the thickness variation due to the short coherence length of the light source, the precision of thin film fabrication is the key to the fabrication repeatability of the sensor. The thickness obtained by the dip coating method is determined by the viscosity of the coating solution as well as the withdrawal speed when removing the fiber from the liquid. By controlling the reaction conditions such as temperature and reaction time, the coating solution with the same properties can be achieved repeatedly. The spectra of 6 sensors are shown in the supplementary file (Fig. S.6) with very similar features demonstrating the fabrication repeatability of the FPI sensor, although the peak positions are not exactly the same due to the small variation of the thickness caused by the dip coating process (the average thickness of 6 sensors is calculated as 5.27 ± 0.4 µm).

The drying process in 100 % N_2_ atmosphere is a critical point behind this as it dramatically improves the hydrophobicity of the sol–gel matrix film and reduces the permeability of water molecules that can interact with the dye’s ion-pair compared to the air dried film [15]. Other drying conditions such as annealing in high temperature may also help to improve the hydrophobicity, but it may risk destroying the base catalyst due to thermal decomposition. On the other hand, the massive collapse of the pores of the matrix film during annealing will lead to the low sensitivity of the target gases due to the low gas permeability. The dye-phase transfer based CO2 sensor typically suffers a short lifetime due to the gradual neutralisation of the pH in the air [15]. Our previous publication reveals that with a proper storage condition such as in a pure N_2_ atmosphere, the lifetime is significantly extended with spectrum stabilised over time [15]. For the reported hybrid CO_2_ and RH sensor, we discovered that preserve the sensor in a vacuum condition such as a vacuum food bag, both absorption and fringes remain close to the freshly prepared sensor even after approximately two years of storage time (shown in Fig. S.7). The intensity fluctuation of the light source could affect the CO_2_ measurement, especially for the long-term measurement, whereas the RH measurement is not affected due to the wavelength shift-based measurement. The light source used in this work is very stable but is costly. Alternatively, the intensity ratio between the signal (intensity value defined in previous) over the reference wavelengths can be applied to compensate for the intensity drift. The reference wavelengths can be outside of the absorption window (i.e. at a wavelength around 700 nm) indicating the drift of light source only, or the intensity around 450 nm which changes in opposite direction to the signal in response to CO_2_.

The developed FOS exhibits comparable performance to the commercial CO_2_ datalogger regarding determining the CO_2_ and RH level from the breath sample but with much smaller sensor size. It is probably the smallest sensor that capable of measuring both CO_2_ and RH compared with the other types of sensors that have been reported [11], [12], [13], [14], [15], [16], [17], [18], [19], [20], [21]. The compact size demonstrates an important advantage of the FOS for application in a confined space and a sealed environment where the FOS can be incorporated into a needle device without breaking the seal. The FOS can be easily incorporated into a ventilation system for supporting endotracheal intubation by detecting CO_2_ expelled from lungs and monitoring the RH delivered to the patient from the ventilation system in the clinical intensive care unit.

## 5 Conclusion

A novel hybrid optical fiber sensor combining a colorimetric sensor and a Fabry–Pérot interferometer for measuring CO_2_ and RH is reported. The sensor is constructed with a single thin film (thickness: ～ 5.9 µm) comprised of thymol blue and TMAH encapsulated Ormosil on the tip of an optical fibre through a dip coating process. The fabrication repeatability of the sensor is demonstrated with a variation in film thickness of about 0.4 µm. The sensor shows a reversible response to CO_2_ and RH for the tested ranges (0–6% of CO_2_ and 0–90% of RH) with the changes in absorption intensity and interference wavelengths, respectively, and with negligible cross-sensitivity between those two measured parameters. The CO_2_ sensitivity of the reported sensor is influenced by the temperature and an increase of temperature (from 20 °C to 40 °C) reduces the sensitivity, and without compensation of temperature, the sensor produces a percent error of < 14% for measuring of 5% of CO_2_ when temperature fluctuation ≤ 5 °C. The measurement of RH is, on the other hand, not affected by temperature. The sensor is demonstrated to measure the CO_2_ and RH simultaneously from human breath with a percent error of 3.1% and 2.2% respectively to the commercial sensor. A simulation model adapted from the conventional FPI model demonstrates the capability of simulating absorption spectra of such sensors with different film thickness.

## Funding

This work was funded by the Medical Research Council (grant number: MR/R025266/1).

### CRediT authorship contribution statement

**LiangLiang Liu:** Conceptualization, Data curation, Formal analysis, Investigation, Methodology, Writing – original draft. **Stephen P. Morgan:** Conceptualization, Funding acquisition, Methodology, Project administration, Resources, Software, Supervision, Validation, Writing – review & editing. **Ricardo Correia:** Conceptualization, Funding acquisition, Methodology, Project administration, Resources, Software, Supervision, Validation, Writing – review & editing. **Serhiy Korposh:** Conceptualization, Funding acquisition, Methodology, Project administration, Resources, Software, Supervision, Validation, Writing – review & editing.

## Declaration of Competing Interest

The authors declare that they have no known competing financial interests or personal relationships that could have appeared to influence the work reported in this paper.
