# Supplementary material for: A single-film fiber optical sensor for simultaneous measurement of carbon dioxide and relative humidity
Source: Opt Laser Technol. 2022 Mar;147:None. doi: 10.1016/j.optlastec.2021.107696 (PMC8689145; doi:10.1016/j.optlastec.2021.107696)
Supplement: Supplementary data 1 [file mmc1.docx]

**A single-film fiber optical sensor for simultaneous measurement of carbon dioxide and relative humidity**

LiangLiang Liu, Stephen P Morgan _,_ Ricardo Correia, Serhiy Korposh^*^

Optics and Photonics Group, Faculty of Engineering, University of Nottingham, University Park, Nottingham, UK, NG7 2RD

* s.korposh@nottingham.ac.uk

Supplementary information

This document is provided as supporting results for the submitted manuscript. Graphs attached in this document are numbered differently to the graphs in the manuscript, and they are referenced with the relevant figure number in the main manuscript where they are first mentioned.

## Materials

Tetraethyl Orthosilicate (TEOS), triethoxymethylsilane (MTEOS), thymol blue, ethanol and tetramethylammonium hydroxide (TMAH) solution (10 wt % in water), Tedlar bag (2L) were purchased from Sigma-Aldrich, UK. Carbon dioxide was purchased from Ryvalgas, UK. Nitrogen (oxygen-free) was purchased from BOC, UK.

1. Sensor Preparation

The sol-gel coating solution is produced by mixing 200 μL of TEOS with 1.8 mL of MTEOS in a 20 mL volume vial under stirring for 10 mins, subsequently adding 5 ml of ethanol and stirring for another 10 mins. Weighing 22 mg of thymol blue powder and added into the mixed solution with additional stirring for 15mins. 500 μL of TMAH is then dropwise added to the prepared solution during stirring, and the colour of the solution can be observed turning from red to yellow to blue. The vial is then capped and stirred for 1.5 hours before dip-coating.

The tip of a 3dB multimode optical fiber coupler (diameter: 62.5/125 μm, F-CPL-M12855, Newport) is mechanically stripped to remove the plastic jacket and expose the silica fiber, then cleaned with isopropanol alcohol and cleaved at 90 degree to create a smooth cutting surface. The tip is then dip into the coating solution for 5 s, then withdraw in a constant speed of 0.5 mm/s by using in-house dip coater to remove the fiber back to air. This procedure is repeated 3 times. A drying period of 2 min between each coating is allowed. The coated optical fiber is then dried in 100% N2 (humidity free) for 24 hours before use. The dried sensor is preserved in a vacuum bag when not in use and stored in a dark atmosphere.

1. The principle of the FPI dye sensor


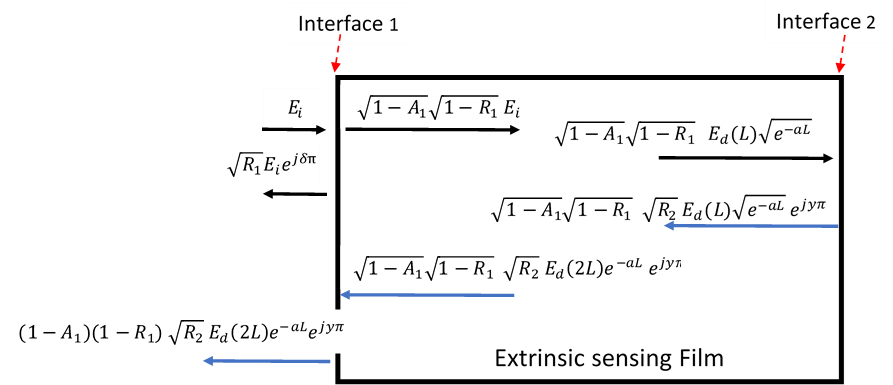


**Fig.S.1** Schematic of the electric field change during propagation.

Due to the high losses within the cavity as absorption and low reflection from interface, the higher order reflections (i.e. multiple reflection) are considered negligible and the system is treated as two beam interference. The input electric field *E_i_* undergoes reflection from the interface 1 (between fibre core and film) and second reflection from the interface 2 (between the film and air) (Fig.S.1). Losses from transmission, reflection, absorption are introduced during the optical pathway with $A_{1}$ is the transmission loss factor of the interface 1, which results from the surface imperfections (e.g. roughness); $a$ is the absorption coefficient of the dye (thymol blue) and is associated with the extinction coefficient ($\varepsilon_{\lambda}$) and the dye concentration ($c$) within the cavity; $R_{1}$ and $R_{2}$ are the reflections from the two interfaces. Besides, δ and y are 1 or 0 respectively as determined by the reflection phase change (i.e. **π** or none) on interface 1 or 2, λ is the wavelength of incident light, *L* is the cavity length and *n* is the refractive index of the cavity material, $K$ is the total loss factor of the sensor head, $\omega_{0}$ is beam waist diameter.

As The electric field coupled back to the optical fibre is then expresssed as:

$E_{r}=\sqrt{R_{1}}E_{i}e^{j\delta\pi}+(1-A_{1})(1-R_{1}) \sqrt{R_{2}} E_{d}\left( 2L \right)e^{-aL}e^{jy\pi}$ (1)

Where $E_{d}\left( 2L \right)$ is the diffracted electric field after path length of 2L. The normalised intensity coupled back to the optical fibre is thus expressed as:

$$I_{r}=\left| \frac{E_{r}}{E_{i}} \right|^{2}=R_{1}+\left( 1-A_{1} \right)^{2}\left( 1-R_{1} \right)^{2} R_{2} \left( \frac{E_{d}\left( 2L \right)}{E_{i}} \right)^{2}e^{-2aL}$$

$+2\sqrt{R_{1}R_{2}}\left( 1-A_{1} \right)\left( 1-R_{1} \right) \frac{E_{d}\left( 2L \right)}{E_{i}} e^{-aL}$ (2)

Some of expression can be obtained from below:

$E_{i}\left( r \right)=\frac{A}{\omega_{0}}\exp\left( -\frac{r^{2}}{\omega_{0}^{2}} \right)$ (3)

$E_{d}\left( r,z \right)=\frac{A}{\omega(z)}\exp\left( -\frac{r^{2}}{\omega^{2}(z)} \right)exp[-jɸ(r,z)]$ (4)

$\frac{E_{d} \left( 2L \right)}{E_{i}}=\exp\left[ -jɸ\left( r,z \right) \right]\left[ 1+\frac{{4L}^{2}\lambda^{2}}{\pi^{2}\omega_{0}^{4}} \right]^{-\frac{1}{4}}$ (5)

$R_{1}=|\frac{n_{2}-n_{1}}{n_{2}+n_{1}}\left. \right|^{2}$ (6)

$R_{2}=|\frac{n_{3}-n_{2}}{n_{3}+n_{2}}\left. \right|^{2}$ (7)

The normalised intensity is then further simplified as:

$I_{r}=R_{1}+K^{2}\left( 1-R_{1} \right)^{2} R_{2} +2K\sqrt{R_{1}R_{2}}\left( 1-R_{1} \right)Cos(\frac{4\pi}{\lambda}nL-arctan(\frac{2\lambda L}{\pi\omega_{0}^{2}}))$ (8)

$K=\left[ 1+\frac{{4L}^{2}\lambda^{2}}{\pi^{2}\omega_{0}^{4}} \right]^{-\frac{1}{4}}(1-A_{1})e^{-aL}$ (9)

$a=2.303c\varepsilon_{\lambda}=2.303A/L$ (10)

where ω_0_, ω(z) = ω_0_(1 + z ^2^/ f ^2^ ) ^1/2^ and φ(r, z) ≈ kz − ϕ(z) are respectively defined as the beam-waist diameter, beam radius and total phase factor; z is the propagation distance along the axis direction of the field from the beam waist located at z = 0 (interface 1), while r is the radial coordinate; A = (2/π) ^1/2^ is the power normalized coefficient with k = 2πn_m_/λ; f = πω_0_ ^2^/λ is the Rayleigh range and ϕ(z) = arctan(z/ f) is the Gouy phase shift. n_1_, n_2_, n_3_ are the refractive index of the fibre core, sensing film and air, respectively.

1. Results


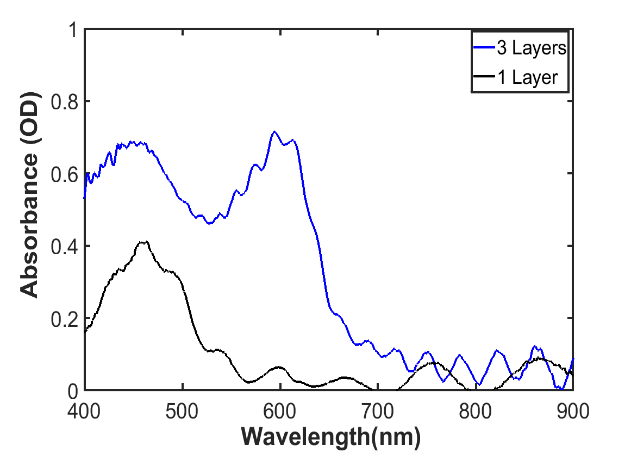


**Fig. S.2** The absorption spectrum of the FOS with 1 layer and 3 layers after drying.


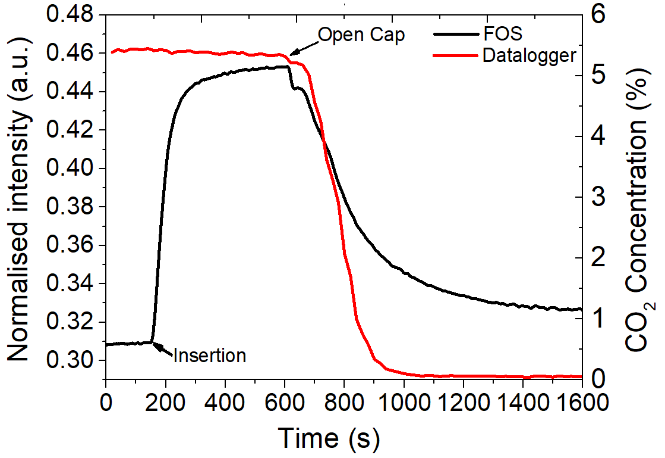


**Fig. S.3** The response test of the FOS to CO_2_. The sensor is inserted into the chamber which is filled up with ~6 % CO_2_


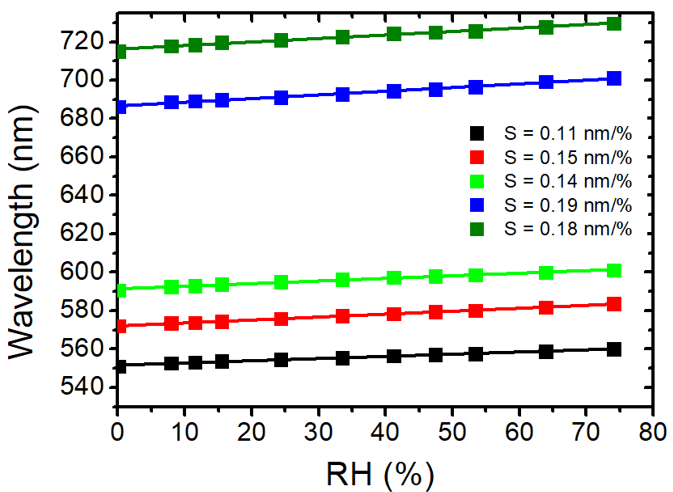


**Fig. S.4** The wavelength of each interference peak as a function of RH.


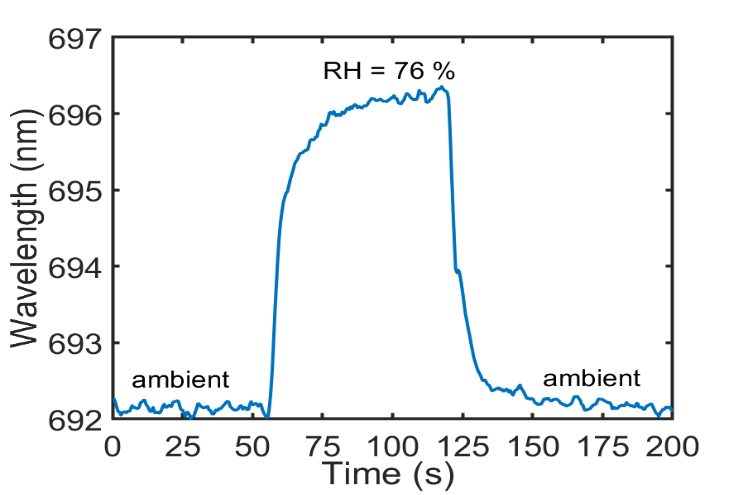


**Fig. S.5** The response time of FOS to RH; the sensor was inserted into the chamber with a humidity level of 76% at the time of 220 s and open the cap at 478s for reducing the RH back to ambient level (33%).


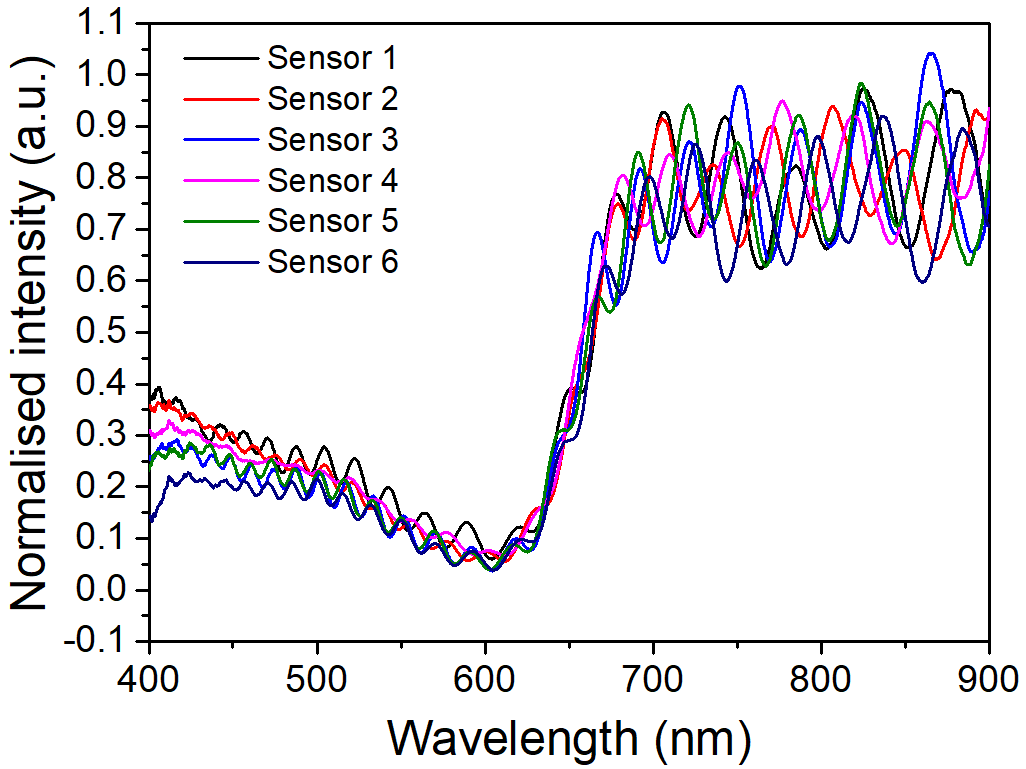


**Fig. S.6** Spectra of the 6 fabricated sensors by using the proposed fabrication method.


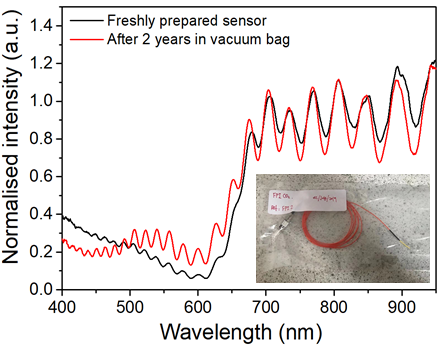


**Fig. S.7** Spectrum of the sensor in freshly prepared (black) and stored in vacuum for 2 years (red). Inset is the fibre sensor sealed in a vacuum food bag.
